# Inhibition of beta-catenin shows therapeutic potential in head and neck squamous cell carcinoma in vitro

**DOI:** 10.1007/s00405-022-07598-y

**Published:** 2022-08-24

**Authors:** Tobias Maier, Stefan Stoiber, Elisabeth Gurnhofer, Markus Haas, Lukas Kenner, Gregor Heiduschka, Lorenz Kadletz-Wanke, Faris F. Brkic

**Affiliations:** 1 Department of Otorhinolaryngology and Head and Neck Surgery, Medical University of Vienna, Vienna, Austria; 2 Department of Pathology, Medical University of Vienna, Vienna, Austria; 3 Christian Doppler Laboratory for Applied Metabolomics, Vienna, Austria; 4 Unit of Laboratory Animal Pathology, University of Veterinary Medicine, Vienna, Austria; 5 CBmed GmbH-Center for Biomarker Research in Medicine, Graz, Austria

**Keywords:** Wnt, Beta-catenin, MSAB, HPV, HNSCC, Cell culture

## Abstract

Beta-catenin is known to be a vital component of the canonical Wnt signaling cascade, involved in the carcinogenesis of different solid tumors. We aimed to evaluate the effects of Beta-catenin inhibition in head and neck squamous cell carcinoma (HNSCC) in vitro. The small molecular compound MSAB was used to inhibit Wnt/Beta-catenin signaling in a human papillomavirus (HPV)-positive and HPV-negative cell line and its effects on cell proliferation, migration, colony formation, apoptosis, as well as radiosensitizing properties were assessed. Significant antineoplastic effects were observed in both cell lines. Interestingly, stronger anti-neoplastic and radiosensitizing effects were observed in the HPV-negative cell line, whereas stronger anti-migratory potential was detected in HPV-positive HNSCC cells. In conclusion, our findings suggest MSAB as a potential therapeutic agent for HNSCC. Further studies are warranted to unravel the mechanistic background of our findings.

## Introduction

Head and neck cancers account for nearly half a million deaths worldwide and about 900,000 new cases each year, which makes it the sixth most common cancer type [1, 2]. Most important risk factors for HNSCC are alcohol, smoking, and HPV infection [3–5]. Due to changes in lifestyle choices and reduced alcohol and tobacco consumption, head and neck cancer incidence rates are declining globally [6]. Nevertheless, HPV-induced oropharyngeal squamous cell carcinoma (OPSCC) is on the rise [7, 8]. Indeed, HPV positivity in HNSCC is associated with better clinical outcome. Based on this, treatment de-escalation is currently being discussed for this patient group [9, 10]. Still, certain patient subgroups are presented with a very poor prognosis [11, 12]. Furthermore, treatment with cisplatin, commonly used in HNSCC, associates with side effects, such as hearing loss, kidney failure, and neuropathy [13]. In addition, resistance to radiotherapy (RT) is another main contributor to patient mortality and morbidity [14, 15]. This warrants the search for new therapeutic agents that target the distinct characteristics of HNSCC and have synergistic effects with RT. The Wnt/Beta-catenin (WBC) signaling pathway is one promising target that has been identified. This signaling cascade is physiologically active during embryogenesis and is responsible for the proliferation, migration, and differentiation of cells. However, its deregulation contributes to carcinogenesis of different solid tumors. In colorectal cancer, the overactivation of WBC and its involvement in cancer development is well-known [16]. Furthermore, deregulation of WBC signaling seems to contribute to leukemia, melanoma, and breast cancer development [17–20]. Recent findings suggest the involvement of the WBC pathway in disease progression in HNSCC [21]. Moreover, HPV seems to be involved in the activation of the canonical Wnt pathway, although its consequences in HPV-positive HNSCC are still unknown [22].

Methyl 3-{[(4-methylphenyl)sulfonyl]amino}benzoate (MSAB) binds to Beta-catenin resulting in its ubiquitination, subsequently promoting its proteasomal degradation and, therefore, inhibiting the WBC pathway-induced gene transcription. MSAB was discovered by Hwang et al*.* in 2016 and showed anti-proliferative effects in Wnt-dependent cancer cell lines. In addition, MSAB showed reduction in the size and weight of Wnt-dependent HCT116, HT115, and H23 xenograft tumors in mouse models [23].

Regarding inhibitors of the WBC pathway, ICG-001 has already been studied for its antineoplastic effects in HPV-negative and HPV-positive cell lines [24]. It showed anti-proliferative and anti-migratory as well as induction of apoptosis and reduction of WBC pathway target genes. ICG-001 inhibits the CREB-binding protein (CBP), which is a transcriptional coactivator and is needed for WBC pathway-dependent gene transcription. Furthermore, PRI-724, an ICG-001 analog, was shown to affect Beta-catenin-dependent gene expression, induce apoptosis and inhibit cell migration in HNSCC cells [25]. However, there is no available data concerning Beta-catenin inhibition by MSAB in HNSCC.

Therefore, in this study, we aimed to investigate the potential antineoplastic and radiosensitizing effects of MSAB in HNSCC cells. The primary aim of the study is to investigate antiproliferation, anti-migratory, pro-apoptotic, and anticlonogenic potential of MSAB in HPV-positive and HPV-negative HNSCC cell lines. The secondary aim was to assess the synergistic potential of the therapy with MSAB with RT.

## Materials and methods

### Cell culture

MSAB was evaluated for its effects in an HPV-negative (Cal 27) and HPV-positive (SCC154) cell line. These were acquired from the American Type Culture Collection (ATCC, Manassas, Virginia, USA) and regularly tested for mycoplasma infection. Cells were cultured in Dulbecco’s modified eagle’s medium (DMEM) (Gibco, Thermo Fischer Scientific, Waltham, Massachusetts, USA) supplemented with 10% fetal bovine serum (FBS) and 1% Penicillin/Streptomycin (P/S) (both Gibco, Thermo Fisher Scientific, Waltham, Massachusetts, USA), hereafter referred to as culture medium. Cells were cultivated in standard Petri dishes (Sarstedt, Nürnbrecht, Germany) in a humidified environment at 37 °C and 5% CO_2_ (Hera Cell 240, Heraeus Holding GmbH). Subculturing of cells was done in accordance with ATCC recommendations, whereat 1 × Dulbecco's phosphate-buffered saline (DPBS) (Gibco, Thermo Fischer Scientific, Waltham, Massachusetts, USA) and a 0.05% Trypsin—0.53 mM EDTA solution (Sigma-Aldrich, St. Louis, Missouri, USA) was used for washing and detaching the cells. 0.4% Trypan Blue Solution (Sigma-Aldrich, St. Louis, Missouri, USA) and the Countess FL automated cell counter (Thermo Fisher Scientific, Waltham, Massachusetts, USA) were used for cell counting.

We acquired MSAB from Selleckchem (Houston, Texas, USA). In vitro experiments were performed at least 3 times (unless noted otherwise) and mean values ± standard deviations (SD) were calculated for statistical analysis and subsequent graphical representation.

### Cytotoxicity assay

First, a dose–response assay with resazurin (Sigma-Aldrich, St. Louis, Missouri, USA) reduction as a readout was conducted with both cell lines. 96-well plates (Sarstedt, Nürnbrecht, Germany) were used and 5000 cells/well for Cal 27 cells and 20,000 cells/well for SCC154 were seeded in 100 µl culture medium. Next, 24 h after seeding, cells were treated with 100 µl of inhibitor. The inhibitor was dissolved in dimethyl sulfoxide (DMSO) (Sigma-Aldrich, St. Louis, Missouri, USA) and diluted in culture medium (inhibitor dose ranges: 0.125–2 µM (Cal 27) and 0.375–6 µM (SCC154); five replicates per dose). DMSO-treated cells served as a vehicle-control. Furthermore, for the co-treatment with RT, cells were additionally irradiated with 2, 4, and 6 Gray (Gy) using a YXLON Maxishot unit (YXLON International GmbH, Hamburg, Germany). After 72 h, the culture medium was removed and replaced with 100 µl of 56 µM resazurin solution. Cal 27 and SCC154 cells were then incubated for 90 and 150 min, respectively. Measurements were performed with the TECAN multimode microplate reader (TECAN Spark, Tecan Group Ltd., Maennedorf, Switzerland).

### Migration

After the dose–response assay, we performed the migration assay. For this, 24-well plates (Greiner Bio-One, Frickenhausen, Germany) were used. To create the initial gap, culture insert wells (Greiner Bio-One, Frickenhausen, Germany) were utilized. In particular, 75,000 Cal 27 or 350,000 SCC154 cells, respectively, were seeded into each side of the culture inserts. Cells reached 100% confluency before insert removal. DMEM with 1% P/S and 1% FBS was used to starve the cells and limit the proliferation. Once the cells reached 100% confluency, the cell culture inserts were removed. Next, the cells were washed with 1 × DPBS and starvation medium containing various concentrations of MSAB (1 and 4 µM for Cal 27; 0.4 and 0.8 µM for SCC154) was added. The first visualization was performed with the TECAN multimode microplate reader immediately after treatment. Further visualizations were performed after 24 and 48 h for SCC154 cells and after 18 h for Cal 27 cells. Prior to each visualization timepoint, cells were washed and fresh starvation medium containing inhibitor was added. Calculation and analysis of the images was done with ImageJ 1.53e using the Wound Healing Tool. [26].

### Colony formation assay

A colony formation assay was used to determine the anticlonogenic potential of the MSAB. As SCC154 cells require cell-to-cell contact to proliferate, the observed results in the clonogenic assay were not reliable nor replicable. The Cal 27 cells were seeded in 12-well plates (Sarstedt, Nürnbrecht, Germany) with a density of 250 cells/well in 1 ml. Twenty-four h after seeding, cells were treated with MSAB at final concentrations of 0.25, 0.5, and 1 µM, or DMSO (all diluted in 1 ml culture medium). The medium was aspirated 72 h after treatment, and 1 ml fresh culture medium was added. 9 days after seeding, colonies of sufficient size formed. To eliminate floating or dead cells, as well as cell debris, wells were washed with 1 ml 1 × DPBS directly before the measurement. The TECAN multimode microplate reader was used to take pictures of the wells and a self-written macro for ImageJ 1.53e.3 was used to count the generated colonies [26].

### Cell apoptosis assay

A caspase-3/7 assay, utilizing the Caspase-Glo 3/7 Assay Kit (Promega, Fitchburg, Wisconsin, USA), was performed to evaluate the pro-apoptotic potential of MSAB. Ninety-six-well plates (Sarstedt, Nürnbrecht, Germany) were used. For this, 5000 cells/well were seeded for Cal 27 cells and 20,000 cells/well for SCC154 in 100 µl culture medium. Then, 24 h after seeding, the culture medium was aspirated and MSAB at various concentrations (0.5 and 1 µM for Cal 27, and 1 and 2 µM for SCC154 diluted in culture medium) was added. Moreover, 40 µM cisplatin served as positive control and DMSO as vehicle-control. For each condition, duplicates were performed. The blanks contained culture medium and MSAB at 2 µM. 100 µl of Caspase-Glo 3/7 Reagent was added 24 h after treatment. Preparation of reagents and workflow was done in accordance with the protocol provided by the vendor. Cells were incubated for 30 min, the supernatant was then transferred to a white, flat-bottomed 96-well plate (Sarstedt, Nürnbrecht, Germany) and the luminescence was measured with the TECAN multimode microplate reader.

### Statistics

Statistical significance for the cytotoxicity assay was assessed by a two-way ANOVA. For the migration, clonogenic, and apoptosis assay, the statistical significance was calculated using a one-way ANOVA. Statistical analyses and graphical presentations were conducted with GraphPad Prism version 8.4.2 for Windows (GraphPad Software, San Diego, California USA, www.graphpad.com). The online tool SynergyFinder (FIMM) was used to evaluate possible synergistic interactions on cell viability between MSAB and irradiation using a zero-interaction potency (ZIP) model [27]. Data is presented as mean values ± standard deviation (SD).

## Results

### MSAB decreases viability in HNSCC cells

**Fig. 1:**
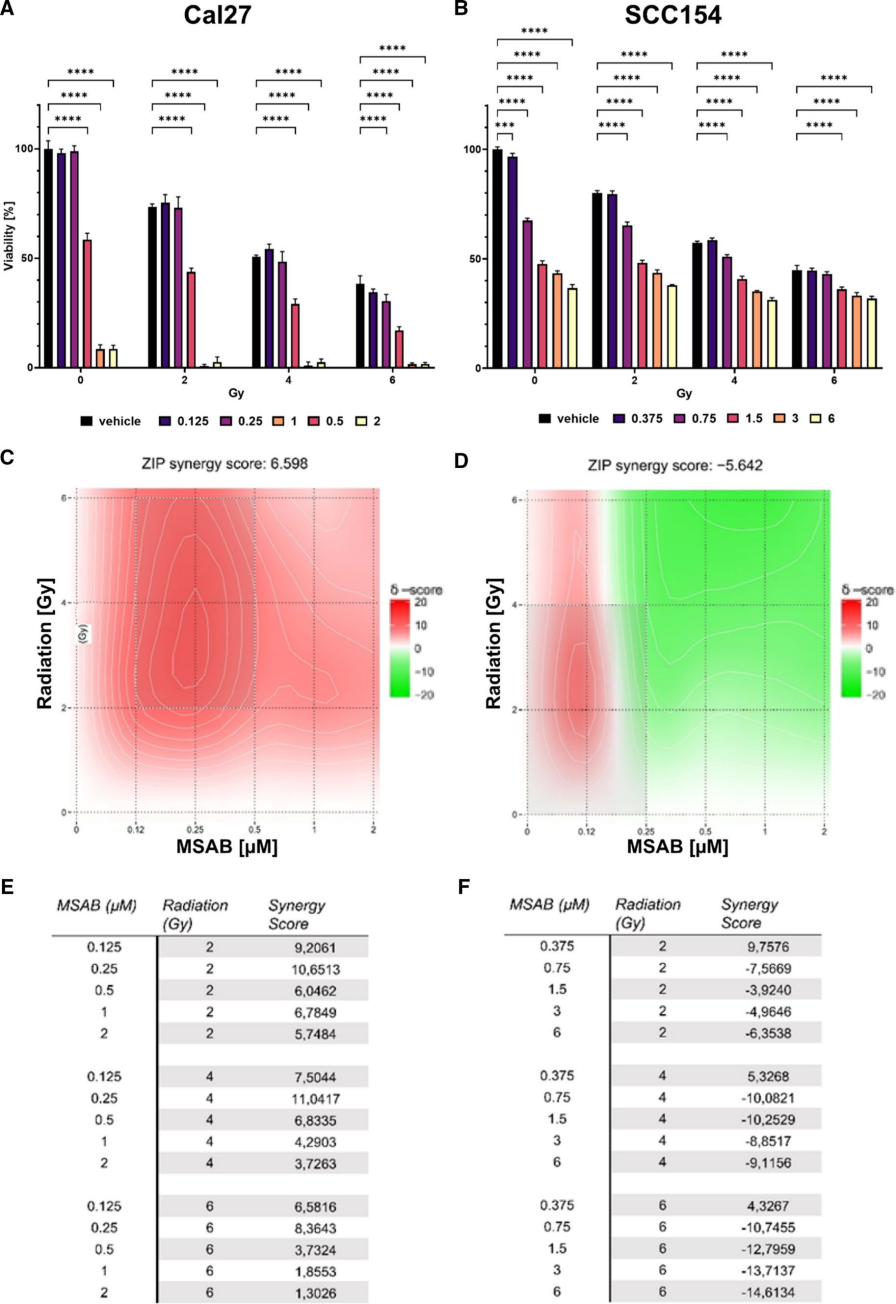
*MSAB decreases cell viability in a dose-dependent manner and synergizes with radiation treatment*. Changes in proliferative ability due to increasing doses of MSAB and radiation in single or combination treatment for Cal 27 (**A**) and SCC154 cells (**B**), 72 h after treatment. Graphs represent mean values ± SD, whereby five replicates were used per dose. Maps of ZIP synergy scores for Cal 27 (**C**) and SCC154 (**D**) at each MSAB and irradiation combination are shown. Tabular results of the ZIP synergy analysis for Cal27 (**E**) and SCC154 (**F**). Synergy is defined as excess response due to drug and radiation interactions, whereby scores higher than 10 can be interpreted as synergistic interactions, scores between 10 to − 10 suggest additive effects, and scores under − 10 indicate antagonistic effects. Significance was calculated via two-way ANOVA and asterisks indicate significant differences between vehicle control and inhibitor concentration in the respective radiation group (***p ≤ 0.001, ****p ≤ 0.0001)

A resazurin reduction assay was conducted to evaluate the effect of MSAB on cell viability. Cal 27 cells were treated with a dose range from 0.125 to 2 µM and SCC154 cells were treated with 0.375 to 6 µM of MSAB. Cells were further treated with a radiation dose of 2, 4, or 6 Gy to assess possible radiosensitizing effects in combination with MSAB. 72 h after MSAB and radiation treatment, cell viability was evaluated. Cell viability was decreased in a dose-dependent manner upon MSAB and radiation treatment (Fig. 1a, b). HPV-negative cells showed a higher sensitivity to MSAB treatment compared to the HPV-positive cells with an absolute IC_50_ of 0.55 µM and 1.34 µM, respectively. Synergy analysis of the effects of concomitant MSAB and radiation revealed overall additive effects were shown for both cell lines, whereat synergistic effects can be observed at a concentration of 0.25 µM MSAB in combination with 2 and 4 Gy in the Cal 27 cell line (Fig. 1c, d). Cell viability data and the zero-interaction potency (ZIP) model were used to calculate synergy scores.

### MSAB shows anti-migratory effects in HNSCC cells

**Fig. 2:**
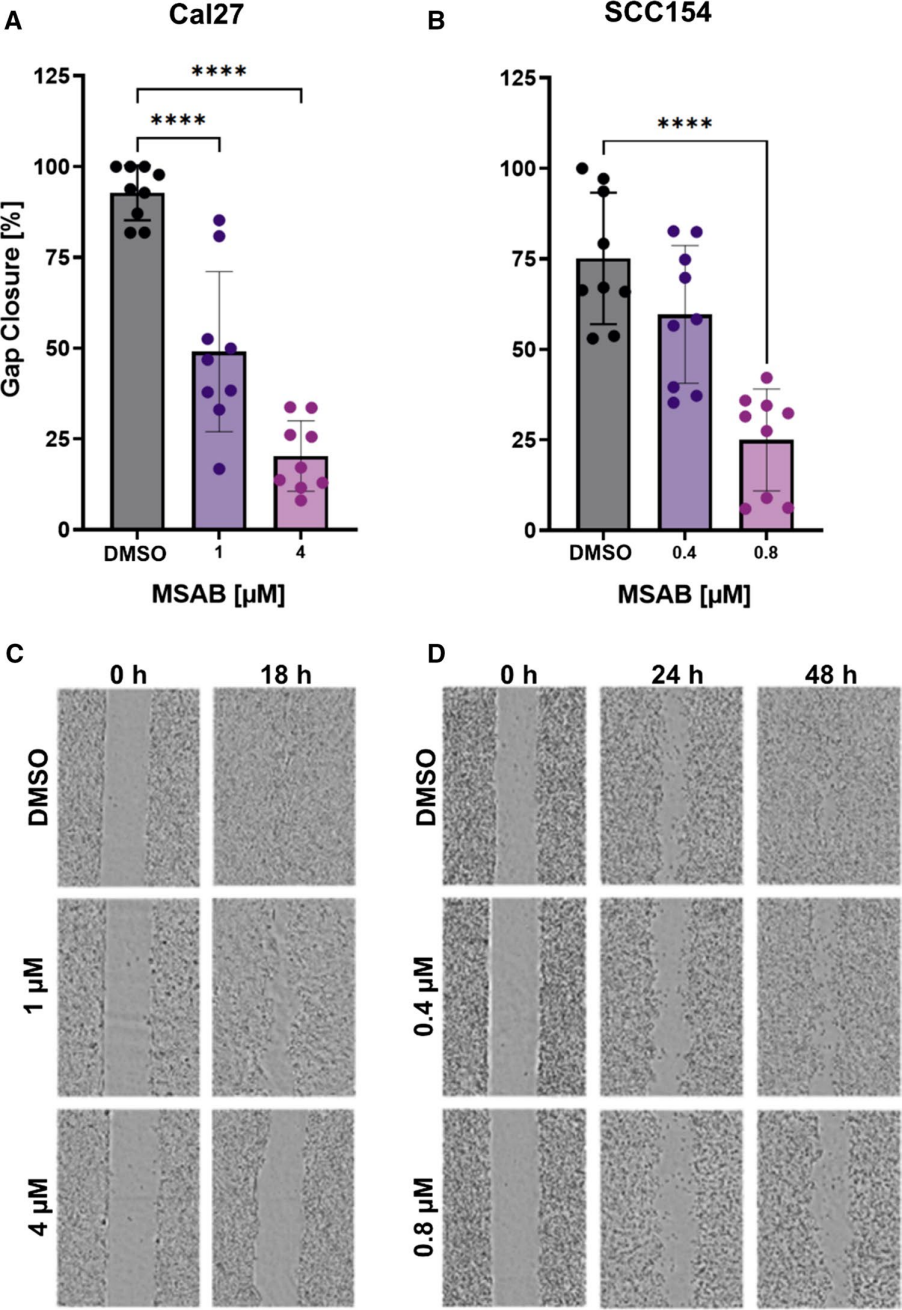
MSAB decreases migratory potential in HNSCC cells. The mean gap closure ± SD is indicated for Cal 27 cells (**A**) and SCC154 cells (**B**) with ascending doses of MSAB. Differences between gap areas were measured between 0 and 18 h for Cal27 and 0 and 48 h for SCC154 cells after gap creation and treatment. Representative pictures are shown in **C** and **D**, respectively. Three independent experiments with three replicates were conducted per dose and statistical significance was assessed via one-way ANOVA. Asterisks represent significant differences between DMSO control and inhibitor treatment (****p ≤ 0.0001)

Effects of MSAB on migratory potential were assessed by utilizing a wound healing assay, whereat Cal 27 cells were treated with 1 and 4 µM and SCC154 cells with 0.4 and 0.8 µM of inhibitor (Fig. 2a, b). ImageJ was used to analyze pictures taken immediately after treatment and after 18 h for Cal 27 cells and 48 h for SCC154 cells (Fig. 2c, d). For Cal 27 cells average gap closure was reduced to 49.0% and 20.3% at MSAB concentrations of1 and 4 µM, respectively, whereas DMSO-treated cells had a gap closure of 92.8% (Fig. 2a, c). DMSO-treated SCC154 cells showed a gap closure of 75.1% compared to 59.6% and 25.0% at MSAB concentrations of 0.4 and 0.8 µM of MSAB, respectively (Fig. 2b, d). Significant reduction in migratory potential was shown for both cell lines, whereat lower concentrations were needed for SCC154 cells to achieve a similar inhibitory effect.

### MSAB decreases clonogenic survival in HPV-negative HNSCC cells

**Fig. 3:**
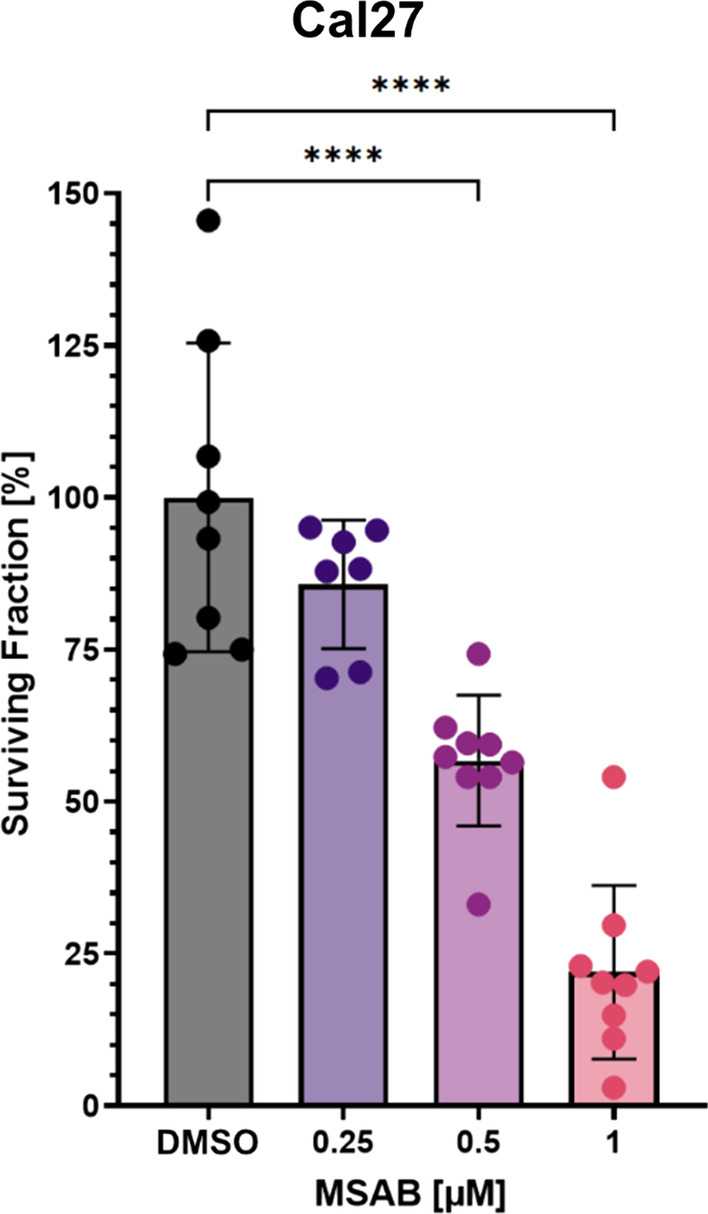
MSAB inhibits clonogenic potential in HNSCC cells. The mean surviving fractions ± SD are shown for Cal27 cells, whereat three independent experiments with two replicates were performed for each dose. Colonies were counted 8 days after treatment with MSAB and DMSO. Surviving fraction was defined as the mean percentage of colonies formed, normalized to vehicle-treated cells. A one-way ANOVA was used for statistical analysis and asterisks represent significant differences between vehicle control and inhibitor-treated cells (****p ≤ 0.0001)

To evaluate the effects of MSAB on clonogenic survival a colony formation assay was performed. Therefore, Cal 27 cells were treated with 0.25, 0.5, and 1 µM of MSAB and vehicle control (Fig. 3). To allow cells to form sufficient colonies, measurement was conducted 8 days after treatment and surviving fractions were calculated. The clonogenic survival of Cal 27 cells was reduced in a dose-dependent manner—the average surviving fraction was reduced to 85.7%, 56.7%, and 22.0% at 0.25, 0.5, and 1 µM of MSAB treatment, respectively (Fig. 3). No reliable results (see methods) were observed in SCC154 cells, probably because of the missing cell-to-cell contact in this assay, which is pivotal for their survival.

### MSAB induces apoptosis in HNSCC cells

**Fig. 4:**
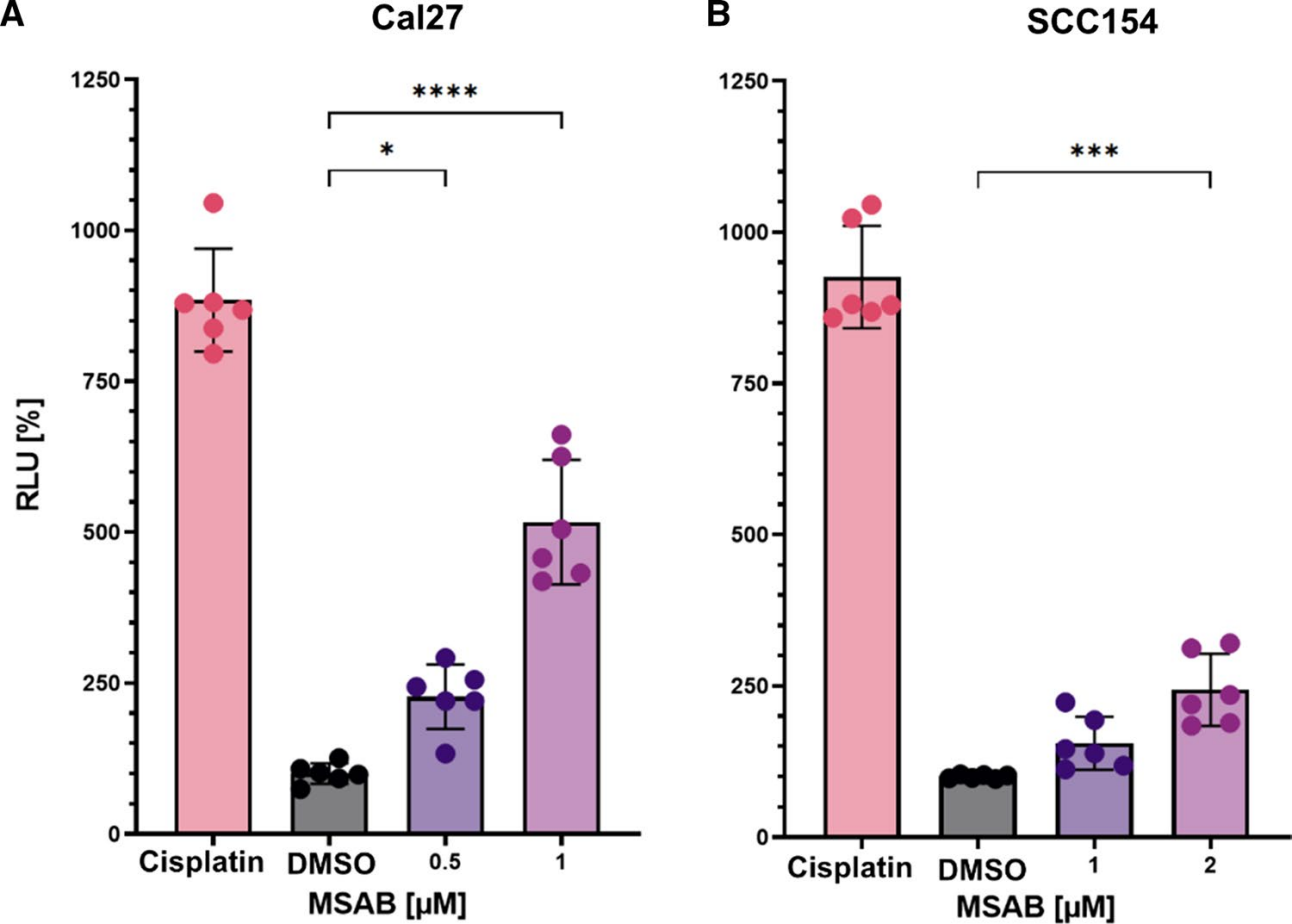
MSAB induces apoptosis in HNSCC cells*.* Cal 27 (**A**) and SCC154 (**B**) cells were treated with ascending doses of MSAB, DMSO, and 40 µM cisplatin, as a positive control, for 24 h before measurement. Graphs represent mean relative light units (RLU) ± SD, normalized to DMSO-treated cells. Cell death was detected via caspase 3/7 activity measurement, whereby three independent experiments with two replicates were performed for each dose. Asterisks represent significant differences between vehicle-control to inhibitor concentration (*p ≤ 0.05, ***p ≤ 0.001, ****p ≤ 0.0001)

Cell death was assessed 24 h after treatment via a caspase 3/7 assay. Doses of 0.5 and 1 µM of MSAB were used for Cal 27 cells and 1 and 2 µM for SCC154 cells. Subsequently, cell death was measured via emitted luminescence of cleaved caspase 3/7-substrate (Fig. 4a, b). Cell The cell death induced via caspase 3/7 was significantly increased to 228% / 517% relative light units (RLU) in treated Cal 27 cells at 0.5 µM and 1 µM MSAB, respectively (Fig. 4a). For the HPV-positive cell line SCC154, 155% and 244% RLU were recorded for MSAB doses of 1 and 2 µM, respectively (Fig. 4b).

## Discussion

As noted, the global rise of HNSCC incidence can be observed, mostly owing to the rising incidence rates of HPV infection in the USA and Europe [7, 8]. Besides the fact that a subgroup of patients with a HPV-positive disease face a poor prognosis [11, 12], there is a lack of substantial improvements in clinical outcome over the last years for HPV-negative HNSCC patients. On the one hand, this underlines the need for identifying of novel therapeutic targets in HNSCC. To achieve treatment de-escalation, the identification and utilization of new molecular targets specific to this patient group are justified. A promising target in HNSCC is the WBC pathway, which was evaluated in this study. Wnt pathway inhibition shows promising results in different cancer types, and a large number of targets were identified in the Wnt signaling cascade [28, 29]. In addition, recent findings suggest that there might be a link between the overactivation of WBC signaling in HNSCC and the HPV oncogenes E6 and E7 [21, 22]. Therefore, our goal was to assess the role of WBC signaling inhibition by the small molecule MSAB in an HPV-positive and HPV-negative HNSCC cell line.

MSAB decreased cell viability in a dose-dependent manner and concomitant irradiation enhanced the anti-proliferative effect in both cell lines. Moreover, radiosensitizing effects of MSAB treatment were observed in the HPV-negative cell line, which is especially vital, since resistance to radiation is one of the most common causes of treatment failure in HNSCC [15]. Synergistic effects were observable at 2 Gy which is also favorable from a clinical standpoint, since sessions with doses of 2 Gy are used in the primary care of HNSCC tumors [30]. These results are in line with other studies, that showed increased cytotoxicity of irradiation in combination with a targeted drug in HNSCC cells [24, 31].

In addition, anti-migratory effects of MSAB treatment were observed in both cell lines. Interestingly, a much lower inhibitor concentration was needed for the HPV-positive cell line to achieve comparable results as with the HPV-negative cells. Furthermore, MSAB treatment impaired colony formation in HPV-negative HNSCC cells. As noted, no viable results could be obtained for the SCC154 cells, since these cells need cell–cell contact to proliferate. Similarly, in lung adenocarcinoma, breast cancer, colorectal cancer, and HNSCC a reduced migratory potential upon Wnt pathway inhibition has been observed in the past [24, 25, 32–34]. In addition, probing for cleaved Caspase 3/7 upon MSAB revealed induction of apoptosis in treated HNSCC cells. These results are coherent with other studies demonstrating the apoptosis-inducing capability of other experimental compounds in HNSCC [25, 35].

Overall, these results showed considerable antineoplastic effects of Wnt pathway inhibition in HPV-negative and HPV-positive HNSCC cells. Better response to MSAB treatment regarding viability and radiosensitizing effects were observed in the HPV-negative cell line, as well as higher rates of apoptosis. These findings are especially pivotal for HPV-negative tumors due to the aforementioned high radioresistance rates. Conversely, better results regarding migration inhibition were shown for the HPV-positive cell line compared to the HPV-negative cells. The inhibition of the Wnt pathway suggests antineoplastic effects in HPV-positive and HPV-negative HNSCC cells, which were also observed in other cancers, where specific inhibitors have already entered clinical phase I and II trials [36–50].

MSAB showed promising antineoplastic effects in-vitro and the underlining mechanism could potentially revolve around the inhibition of the epithelial-to-mesenchymal transition (EMT). Indeed, Liu et al. observed the inhibition of EMT via blockage of the WBC in lung cancer [51]. Some evidence on this interplay were also provided for HNSCC. In particular, the co-expression of Beta-catenin and E-Cadherin, a protein involved in EMT, was associated with clinicopathological parameters in laryngeal squamous cell carcinoma [52]. On the contrary, Greco et al. showed that high expression of Beta-catenin was associated with better survival, while E-Cadherin overexpression was an independent risk factor for worse survival outcome [53]. In total, the interplay of EMT and WBC remains unclear in HNSCC, particularly with regards to MSAB-induced antineoplastic effects, and certainly warrants further investigations.

Our study provided some new and interesting, although only preliminary results. An important limitation of this study is the use of only two cell lines. Therefore, additional HPV-negative and HPV-positive HNSCC cell lines should be included to confirm our findings. Furthermore, different timepoints were used in the migration assay, which reduces the comparability of the results from the two cell lines. Nevertheless, a significant anti-migratory effect was observed in both cell lines. In addition, 3D cell assays may give better and more comparable results to physiological conditions. Finally, future studies should provide insights into the mechanistic nature of MSAB treatment in HNSCC. Nevertheless, we provided strong evidence of the effectiveness of MSAB in HPV-positive and HPV-negative HNSCC in-vitro. Therefore, our findings could potentially serve as a basis for further studies evaluating the therapeutic potential of MSAB in HNSCC.

The diagnostic and prognostic potential of the WBC pathway in HNSCC is well-established and was shown specifically for HPV-positive disease previously by our group [24]. Indeed, we observed better prognosis in patients with a high expression of a WBC-specific protein. Our current study adds further value in terms of the therapeutic potential of WBC inhibition in HNSCC, as we were able to observe significant antineoplastic effects of MSAB in HPV-associated and HPV-negative HNSCC cell lines in-vitro. Certainly, further studies need to be conducted to validate these preliminary findings.

## Conclusions

In the current study, we were able to show antineoplastic effects of Beta-catenin inhibition in HPV-positive and HVP-negative HNSCC cell line models. As these are preliminary, in-vitro results, further validation studies are certainly necessary. Nevertheless, we provided first insights into the therapeutic potential of MSAB in HNSCC. Indeed, MSAB showed anti-proliferative, anti-migratory, and anti-clonogenic effects, as well as apoptosis-inducing and radio-sensitizing properties. Overall, the effect of WBC pathway inhibition by MSAB seems to be higher in Cal 27 cells. These findings suggest MSAB as a potential therapeutic agent in HPV-positive and especially HPV-negative disease, whereas MSAB might serve as a potent inhibitor of migration and, therefore, metastases in HPV-positive HNSCC. Besides validation of our preliminary results, further research is warranted to determine the underlying mechanistics of MSAB treatment as well as to assess its antineoplastic effects in vivo.
